# High performance surface-enhanced Raman scattering substrates of Si-based Au film developed by focused ion beam nanofabrication

**DOI:** 10.1186/1556-276X-7-399

**Published:** 2012-07-17

**Authors:** Tingting Gao, Zongwei Xu, Fengzhou Fang, Wenlong Gao, Qing Zhang, Xiaoxuan Xu

**Affiliations:** 1 State Key Laboratory of Precision Measuring Technology & Instruments, Centre of MicroNano Manufacturing Technology, Tianjin University, Tianjin 300072, China; 2 Tianjin MicroNano Manufacturing Tech. Co., Ltd, TEDA, Tianjin 300457, China; 3 Institute of Physics, Nankai University, Nankai, Tianjin, 300071, China

**Keywords:** SERS, Si-based Au nanostructures, Focus ion beam nanofabrication, Thermal evaporation

## Abstract

A novel method with high flexibility and efficiency for developing SERS substrates is proposed by patterning nanostructures on Si substrates using focused ion beam direct writing (FIBDW) technology following with precise thermal evaporation of gold film on the substrate. The effect of SERS on the substrate was systematically investigated by optimizing the processing parameters and the gold film thickness. The results proved that small dwell time could improve the machining accuracy and obtain smaller nanogap. The Raman-enhanced performance of the substrate was investigated with 10^−6^mol/L Rhodamine 6 G solution. It was indicated that the elliptic nanostructures with 15-nm spacing on Si substrates, coated with approximately 15-nm thick gold film, have exhibited a high-enhanced performance, but dramatic performance degradation was found as the gold film thickness further increased, which most probably resulted from changes of the nanostructures’ morphology such as elliptical tip and spacing. To avoid the morphological changes effectively after depositing gold film, optimization design of the nanostructures for FIBDW on Si substrates was proposed. Besides, a similar phenomenon was found when the gold film was less than 15nm because there was little gold remaining on the substrate. The method proposed in this paper shows a great potential for the higher performance SERS substrates development, which can further reduce the spacing between hot spots.

## Background

Surface-enhanced Raman scattering (SERS) is one of the most powerful tools for trace detections and biochemical applications because of its ultrasensitivity, low-cost, and real-time characteristics [1-6]. In 1928, Raman and Krishnan first observed a special phenomenon that monochromatic light incident on molecules resulted in normal Rayleigh scattering as well as modified scattered radiation of different frequencies. This ‘feeble’ phenomenon is known as Raman scattering, which is attributable to the excitation (or relaxation) of vibration modes of a molecule. Therefore, the Raman spectrum could be used to identify the target molecules up to single molecule in chemical and biological systems [4] because every molecule has its unique Raman spectrum, and different functional groups have different characteristic vibration energies. But in a long time after the Raman scattering was discovered, the applications in biosensing had been limited by its inherent much weak signal until 1977, when Jeanmaire and Van Duyne indicated that the magnitude of Raman scattering signal can be largely enhanced by roughened noble metal surface [7,8]. Later, this phenomenon was defined as SERS [7]. The main underlying enhancement mechanism was attributed to the localized surface plasmon resonance (LSPR) that electronic collective oscillation [9] contributes to the electromagnetic enhancement [10], occurring when the nanostructure is much smaller than the excitation wavelength. The chemical mechanism would also play a role in SERS enhancement with less contribution than LSPR [11].

The most critical aspect of SERS is the research of efficient SERS-active substrates, such as nanostructured surface or nanoparticles of noble metals with suitable physical parameters such as their material, size, shape, and spacing [12,13]. Generally, Ag and Au nanoparticles are regarded as one of the best candidates for SERS substrate studies [14]. During its development, many methods were put forward to fabricate SERS-active substrates, such as roughened electrodes, noble metal colloidal nanoparticles, silver island films, metal films over nanostructured surfaces, acid-etched metal foils, and lithographically produced nanoparticle arrays [15-17]. Nevertheless, fabrication of SERS substrates with both high sensitivity and high stableness remains difficult, and it is costly for routine SERS detection.

**Figure 1:**
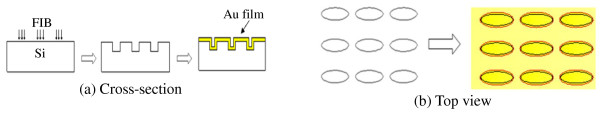
**Schematic images of the process of FIB pre-milling with the following thermal evaporation. (a)** Cross section of the nanostructures before and after thermal evaporation. We can clearly find that the spacing becomes smaller after depositing gold film; **(b)** top view of the nanostructures. It describes the change of the nanostructures’ morphology.

Recently, focused ion beam direct writing (FIBDW) technology has been an increasingly important nano-fabrication technique, which has been used in the SERS substrate’s development [4,10]. However, thin gold film which is coated on the Si substrate or quartz glass is soft, so it has unstable properties during the FIB processing. It is difficult to obtain nanostructures with spacing less than 20nm [18]. Owning to this, we report a simple method for fabricating Au elliptical nanostructures on Si substrates with spacing less than 15nm. The fabrication procedure of the patterned Au nano-ellipse is illustrated in Figure 1. Firstly, we etch nanostructures on Si substrates due to its better fabrication accuracy than that of Au. Secondly, Si substrates were coated with gold film through thermal evaporation with precise film thickness. From Figure 1, we could find that the spacing of the nanostructure became smaller after thermal evaporation. The micrographs of the nanostructures were characterized by scanning electron microscopy (SEM); the thickness of gold film was measured by white light interferometry; and the SERS properties were detected using low concentration Rhodamine 6 G as probe molecules. The result indicates that this nanostructure showed high Raman enhancement with the gold film of approximately 15nm. Moreover, it can be concluded that the edge and spacing are truly the dominant factors for electric enhancement.

## Methods

### Materials and instruments

In this work, Si wafers were cut into squares, cleaned in an ultrasonic bath with methanol for 20min, and dried in the air. Rhodamine 6 G was diluted to 10^−6^mol/L with deionized water. The adsorption peak of R6G molecule in deionized water was 557nm [19], and fluorescence wavelength was 610nm. Therefore, in the experiment, a laser wavelength of 785nm was chosen so as to avoid fluorescence.

FIB system (Nova 200, NanoLab, MA, USA) was used to fabricate patterning nanostructures. It combines ultra-high resolution field emission SEM and precise focused ion beam etch, and could be used for nanoscale prototyping, machining, and on-line high resolution SEM measurements [20]. The thickness of gold film was measured by white light interferometry (NT 9300, Veeco Instruments Inc., Shanghai, China). Raman spectra were obtained with an inVia Raman microscope (Renishaw plc, Gloucestershire, UK) with a CCD detector and a × 50 objective measuring the probe molecules. The nanostructure SERS substrate, which was placed into the R6G solution for 2h and dried in the air, was irradiated by 785nm wavelength with a laser power of approximately 70mW and integration time of 10s. The Raman shift ranges from 550 to 2,000cm^−1^. All tests were carried out on three different places from the substrate, and the final spectra were averaged by those measurements.

### Nanostructures developed on Si substrates by FIB

**Figure 2:**
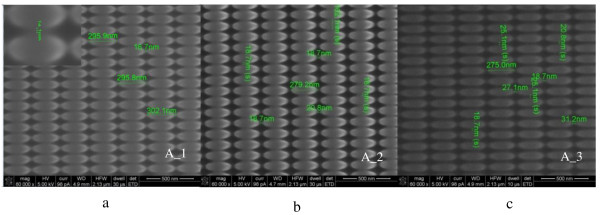
**Scanning electron micrographs of elliptical nanostructure on Si substrates.** The spacings of the elliptical nanostructure were (**a**) 15 ± 1nm, (**b**) 18 ± 1nm, and (**c**) 22 ± 2nm. With the dwell time and etching time increasing, the spacing increased too. Bar = 500nm.

Firstly, ellipse patterns of BMP files with 24-bit format used for the FIB fabrication were compiled with Matlab. In the experiment, the FIB etching size and depth were 9.5 × 9.6μm^2^ and approximately 30 to 40nm, respectively. By adjusting the FIBDW key fabrication parameters, such as dwell time and etching time, micro/nanostructure arrays with high form accuracy were fabricated on Si substrates. Table 1 shows the FIBDW relevant parameters for different substrates, where the average dimensions of the ellipses were 300nm in long axis and 100nm in short axis. Figure 2 shows the SEM images of nanostructures on the Si substrate. The nanostructure spacing can be down to 15nm on Si substrates.

**Table 1:** Description of the dwell time, etching time, and spacing

**Based FIB Si**	**FIB machining parameters**	**Dwell time (μs)**	**Etching time (min)**	**Spacing (nm)**
A_1	30 kV voltage and 10 pa accelerate current	1	8	15 ± 1
A_2		1	11	18 ± 1
A_3		5	8	22 ± 2

### Preparation of SERS-active substrates via thermal evaporation

**Figure 3:**
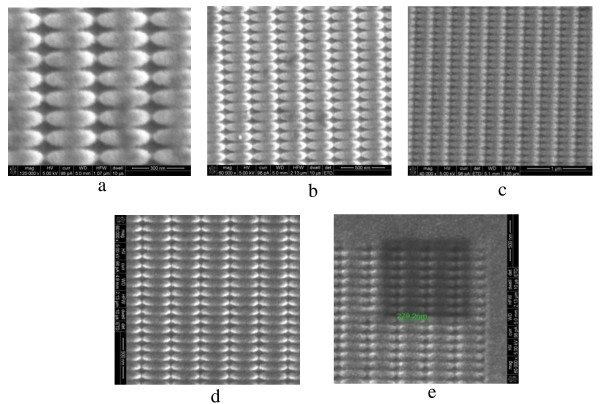
**SEM images of elliptical nanostructures coated with Au of different thicknesses.** (**a**) 10nm, bar = 300nm; (**b**) 15nm, bar = 500nm; (**c**) 18nm, bar = 1μm; (**d**) 20nm, bar = 500nm; and (**e**) 70nm, bar = 500nm.

**Figure 4:**
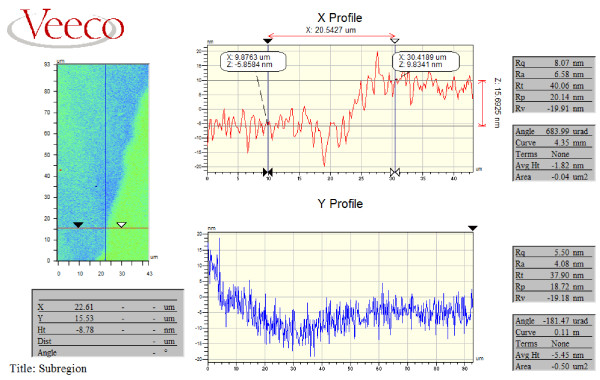
**The thickness of the gold film measured by white light interferometry.** The thickness was about 15nm with deviation of ±2nm.

The SERS-active substrates were made by depositing Au with thermal evaporation. Figure 3 shows the SERS-active substrate with different gold film thickness, and all the substrates were fabricated with a dwell time of 1μs and total time of 8min. In order to estimate the influence of gold film, the thicknesses of the film varied from 10 to 70nm, which were calculated by white light interferometry, as shown in Figure 4. We should note that all the measured thicknesses have a deviation of ± 2nm due to the inaccuracy of the measuring instrument.

## Results and discussion

### Effect of FIB fabrication conditions on SERS

By optimizing the FIBDW parameters, the nanostructure’s dimension and form accuracy on Si substrates can be well controlled, and the nanostructure’s spacing can be reduced to 15nm, as shown in Table 1 and Figure 2. However, bigger dwell time and longer FIB etching time result in apparent changes of the nanostructure’s profile. For example, compared to A_1, the nanostructure’s spacings of A_2 and A_3 increased by 20% and 46.7%, respectively, and the curvature of the elliptical tip becomes bigger. It can be clearly seen in Figure 2. This phenomenon could be attributed to the ion beam etching. Longer FIB fabrication time can induce large-depth structures, but it would also degrade the nanostructures accuracy accordingly.

**Figure 5:**
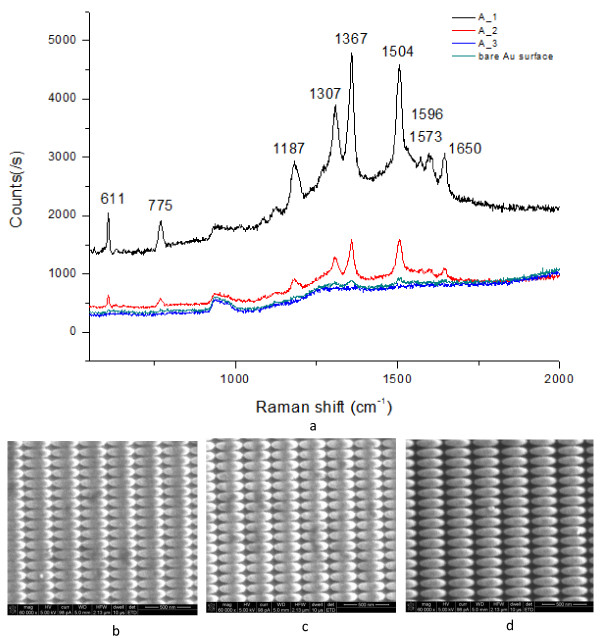
**SERS spectra of 10**^**−6**^ **mol/L R6G for the parameter-dependent substrates.** (**a**) SERS spectra, (**b**) micrographs of A_1 with 15-nm gold film, (**c**) micrographs of A_2 with 15-nm gold film, (**d**) micrographs of A_3 with 15-nm gold film. Bar = 500nm.

In order to test the FIB fabrication accuracy on the SERS enhancement results, the FIB parameter-dependent Si-based substrate with approximately 15-nm thick gold film and neat gold film without nanostructures was characterized by 10^−6^mol/L R6G solution. The substrate was placed into the R6G solution for 2h and dried in the air, then measured by SERS detections. Figure 5 clearly shows the SERS spectra and the related micrographs of the nanostructures after coated with 15-nm thick gold film. The Raman spectrum shows the intense peaks of R6G ’s main vibrational features at 611, 775, 1187, 1307, 1367, 1504, 1573, 1596, and 1650cm^−1^. It is noted that A_1 substrate shows higher Raman signal enhancement than the one for A_2. With increasing dwell and etching times, the Raman signal decreased as shown in Figure 5. It is implied that dwell and etching times were the vital factors for the nanoscale fabrication controlled by FIB. It is worth noting that it was difficult to distinguish any Raman signal from the naked Au surface, which indicated the gold film surface was smooth and homogeneous enough. The high SERS activity in A_1 was mainly attributed to the availability of much smaller structure’s spacing. It is reasonable that the tip gathered amount of charge so that strong electromagnetic field (EM) coupling would be induced by adjacent metal nanostructures when the spacing between two micro/nanostructures decreased [2,21]. The EM effect increases rapidly, which would enhance the signal intensity of SERS.

Therefore, in order to obtain high performance and high electromagnetic field enhancement Si-based gold film SERS substrates, FIB fabrication parameters should be optimized.

### Effect of Au film thickness on SERS

**Figure 6:**
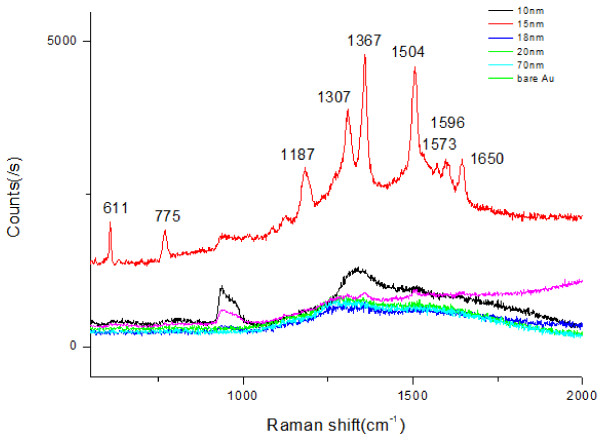
**SERS spectra of 10**^**−6**^ **mol/L R6G for the substrates coated with different film thicknesses.**

For the evaluation of the different film thickness on the quality of SERS spectra, R6G was also chosen as the probe molecule (detection method is the same as the previous work). Obviously, Figure 6 shows the values of Raman signal intensity which almost exponentially decreased with an increasing thickness of the Au film. The elliptical nanostructures coated with approximately 15-nm Au have exhibited very strong SERS effect. The thinner the Au films were, the higher the signal occurred. However, we could not detect any R6G signal when the gold film was 10nm because there was little Au remaining on the surface of the substrate as well as on the sidewall and bottom of the trench. It should also be mentioned that there were no Raman peaks on the bare Au film, noting that the gold film surface was smooth and homogeneous enough. According to Figure 3, we know that when the thickness of Au film is 15nm, the structure form keeps very well, but the FIBDW nanostructures deformed with the thickness increasing to 20 or 70nm, such as the curvature of the elliptical tip and dimension becomes bigger, because the deposition of Au may cover this region. According to the lighting rod effect, geometries with a small curvature lead to charge oscillation and induce a higher EM effect [19,22]. Therefore, the decreased intensities from 15 to 70nm are most probably related to the decreased number of hot spots [23,24] due to the longtime deposition of Au. Since the spacing becomes smaller with the gold film increasing (Figure 1), why was there no Raman signal? It is found that nanostructure tip almost disappeared during deposition progress which obstructed the electromagnetic field enhancement. It can be concluded that, for the geometrical factors of micro/nanostructures, small curvatures and small spacing, which played a key role in promoting cooperative plasmon mode, were equally significant for the creation of a higher electromagnetic field enhanced effect and lead to higher SERS.

### Optimization of the nanostructures

**Figure 7:**
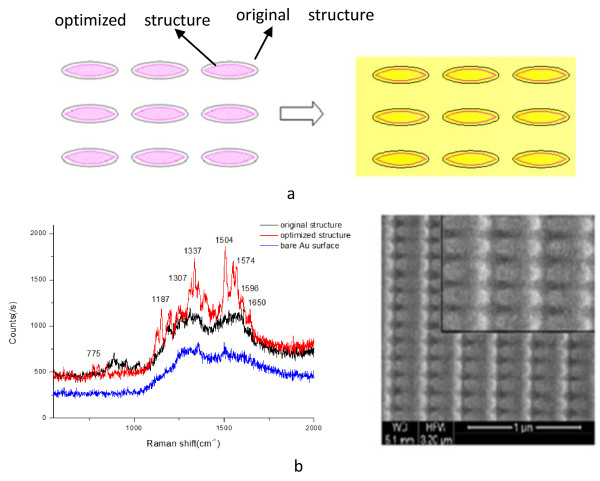
**Schematic representations of the optimization process and Raman spectra after the optimization.** (**a**) Schematic representation of the optimization process. The left represents the contrast of the bitmap files before and after optimization; the right shows the optimized result after depositing gold film. (**b**) Raman spectra after the optimization. The thickness of gold film was 18nm; the sample probed was 10^−6^mol/L R6G.

In order to avoid the performance degradation which was induced by Au film thermal evaporation process, an optimization design method for FIB nanofabrication was proposed. Firstly, the size of nanostructures was minimized during FIB manufacturing process. Secondly, the nanostructures with a smaller curvature should be produced. The bitmap files for FIB fabrication were modified as follows (Figure 7a): the pink area represents the revised structure, where the curvature and size of the elliptical tip become smaller. With an optimized geometry and dimensions, the relative surface area coated with a gold film of 18nm exhibits evident R6G Raman signal (Figure 7b) compared to the original area. The black line represents the original structure; red line, the optimized structure; and blue, the bare Au film. The picture on the right was the optimized structure and the original structure (inset). It is further confirmed that the edge and spacing are the main enhancement factors for SERS. The improvement in the variability of the SERS signal from these optimization substrates indicates that this fabrication process has a great potential for fabricating SERS substrates.

## Conclusions

This paper presents a high enhancement SERS substrate development method with FIB nanofabrication in advance and subsequently with gold film thermal evaporation. It is found that acceleration current, dwell time, and etching time play a major role in precisely controlling nanostructures during the FIB nanofabrication on Si substrates. SERS spectra study revealed the relativities between Raman enhancement and thickness of gold film. As the gold film increased from 15 to 70nm, the Raman signal decreased and disappeared finally because thermal evaporation has covered many hot spots. It is concluded that the spacing and curvature of structures are the key factors for the electromagnetic field enhancement and SERS performance. The method integrated the advantages of high sensitivity and repeatability, and would significantly facilitate practical SERS substrate preparation.

## Competing interests

The authors declare that they have no competing interests.

## Authors' contributions

TG conceived of the study and carried out the fabrication of nanostructures and optimization design. QZ participated in the thermal evaporation of gold films. XX participated in the measurement of gold film. TG and WG participated in the SERS spectra analysis and discussion. ZX and FZF are the advisors of the project, participating in the design of the study, revising the manuscript, and conducting coordination. All authors read and approved the final manuscript.

## Authors' information

FF is a professor working in Tianjin University from 2005. His main research interests are micro/nano manufacturing and freeform optics. He is the president of the International Society for Nanomanufacturing (ISNM). He is also a Fellow of CIRP - International Academy for Production Engineering, and an Editor-in-Chief of the International Journal of Nanomanufacturing (IJNM).
